# Comprehensive analysis of transcriptome data and experimental identification show that solute carrier 35 member A2 (SLC35A2) is a prognostic marker of colorectal cancer

**DOI:** 10.18632/aging.205145

**Published:** 2023-10-26

**Authors:** Yue Wang, Liang Chen, Jing Chen, Zhenzhen Bai, Liyu Cao

**Affiliations:** 1 Department of Pathology, School of Basic Medical Science, Anhui Medical University, Hefei, Anhui, People’s Republic of China; 2 First Hospital of Jiaxing, Affiliated Hospital of Jiaxing University, Jiaxing, Zhejiang, People’s Republic of China; 3 Department of Pathology, Fuyang Hospital Affiliated to Anhui Medical University, Fuyang, Anhui, People’s Republic of China

**Keywords:** SLC35A2, colorectal cancer, prognosis, biomarker, tumor-infiltrating immune cells

## Abstract

Background: Colorectal cancer (CRC) is a solid tumor with high morbidity and mortality rates. Accumulating evidence shows that the soluble carrier family 35 member A2 (SLC35A2), a nucleotide sugar transporter, plays a key role in the pathogenesis of various tumors. However, its expression and function in CRC has not been fully elucidated.

Methods: The prognosis-related gene SLC35A2 was obtained using differential analysis, prognosis correlation analysis, and LASSO regression screening. Its expression levels in CRC tissues were analyzed, and so was the relationship of this expression with clinical characteristics of patients. Subsequently, the expression levels were correlated with clinicopathological parameters using immunohistochemical analysis. Analysis based on GO/KEGG databases was used to reveal the potential mechanisms of SLC35A2. Next, we explored the relationship between SLC35A2 and immune cells in CRC tissues. A nomogram was created to help understand the prognosis of CRC patients. Finally, western blotting and qRT-PCR reaction were used to verify the expression of SLC35A2 in CRC cell lines.

Results: SLC35A2 expression was upregulated and related to tumor pathological stage and lymph node metastasis, indicating that SLC35A2 is an independent prognostic factor and a potential diagnostic marker for CRC. We verified by IHC, WB and PCR that the expression of SLC35A2 was up-regulated in colorectal cancer tissues and cell lines, and its high expression was related to the tumor pathological stage of CRC clinical samples.

Conclusions: Our study found that SLC35A2 can be used as a biomarker for the diagnosis and prognosis of CRC, providing motivation for further study.

## INTRODUCTION

A global public health problem, colorectal cancer (CRC) continues to affect millions of people each year. Due to lifestyle changes, the incidence of CRC is rising worldwide [1]. The latest global data released in 2020 by the World Health Organization’s International Agency for Research on Cancer indicate that colorectal cancer is now the third most common cancer in the world after breast and lung cancers. Among the total new cases of malignant tumors, the proportion of patients with CRC was as high as 10% (1,931,590 cases) [2]. The death rate is second only to lung cancer at 9.4% (935,173 cases) [3]. There are more than 1.06 million men and 860,000 women diagnosed with CRC in the world, with the proportion of new cases in men and women being 10.6% and 9.2%, respectively, ranking third among male cancer cases and second among female cancer cases worldwide [4]. The vast majority (98%) of CRCs are adenocarcinomas. These almost always appear as adenomatous polyps, which can usually be cured by resection [5]; however, early diagnosis and prognosis of CRC is still one of the main challenges facing the medical community. Carcinoembryonic antigen (CEA) is one of most familiarly used serum biomarkers to diagnose colorectal cancer [6]. Nevertheless, the lack of sensitivity and specificity makes CEA detection in early diagnosis ineffective. Gastrointestinal inflammation or other systemic tumors, such as breast and lung cancers, can also lead to an increase in serum CEA levels [7, 8]. The survival rate for patients with colorectal cancer stages I and II was 91% and 82%, respectively, only 12% of patients diagnosed with stage IV disease survive [9]. It is therefore very important to find new effective biomarkers that are important for the early diagnosis and improved prognosis of patients with CRC.

Over 400 membrane-bound proteins make up the human solute carrier (SLC) superfamily, which is crucial for a variety of physiological and pharmacological processes [10]. According to sequence similarity and the expected or observed number of transmembrane α-helices, they are further divided into 65 subfamilies [11]. Nucleoside-sugar compounds are transported by SLC35, a subfamily of nucleotide sugar transporters (NSTs) [12]. The transport of nucleotide sugars from the cytoplasm to the endoplasmic reticulum/Golgi compartment is mediated by nucleotide sugar transporters [13]. In the human genome, 31 members of the SLC35 family have been identified, divided into seven subfamilies-SLC35A-G [14]. Uridine diphosphate (UDP)-galactose is transported through a multi-channel membrane protein encoded by SLC35A2 [15]. There is evidence that many cancers affect SLC35A2’s occurrence and development as a NST member [16–22]. Previous studies have reported that SLC35A2 participates in promoting the metastasis of hepatocellular carcinoma by regulating cell glycosylation, and it’s up-regulation is consistent with the enhanced metastatic ability of HCC cells and the poor prognosis of HCC patients, which may become a potential intervention target for the treatment of advanced HCC in the future [17]; In breast cancer tissues, SLC35A2 is significantly upregulated [18], possibly contributing to breast cancer inflammation and diagnosis [19]. Its expression level is related to the increase of migration and invasion ability of breast cancer cells [20], which is related to the low recurrence-free survival rate. SLC35A2 can be used as an independent prognostic indicator of breast cancer patients [21] and a possible biomarker in immunotherapy [22]. Since SLC35A2’s involvement in CRC hasn’t been fully studied, its clinical application value and molecular mechanism remain unknown.

At present, high-throughput sequencing makes public databases rich in datasets that can be accessed and used. These datasets provide us with an opportunity to further elaborate on the role of oncogenes and better understand possible biological pathways. In the present study, we used several available online databases (UCSC, TGCA, GO/KEGG, and GEO, among others) to perform analyses of expression, prognosis, and immune infiltration to evaluate the clinical value of SLC35A2 for CRC, identify the expression of SLC35A2 in clinical samples and cell lines of CRC patients and investigate its potential origin. Our results provide a reference for the diagnosis, treatment, and prognosis of CRC.

## MATERIALS AND METHODS

### Transcriptome data download and processing

The UCSC database (https://xena.ucsc.edu/) contains data from the TCGA, TARGERT, and other databases. Using this, transcriptome (GDC TCGA Colon Cancer, GDC TCGA Rectal Cancer) and clinical data of CRC were downloaded. Matching allowed the acquisition of 51 normal samples, 616 colon cancer samples, and 584 samples with both expression matrix and clinical data. The transcriptome data download types were HTSeq-FPKM and HTSeq-Counts, in which HTSeq-Counts were used for differential analysis between normal and tumor tissues, while HTSeq-FPKM data were used for subsequent analysis. Log2 was used to transform transcriptome data for later analysis.

### SLC family gene download

We downloaded 450 genes related to the SLC ion channel family from the Bioparadigms website (http://slc.bioparadigms.org) and performed subsequent analysis.

### Difference analysis

In this study, the “Deseq2” package was used, and the HTSeq-Counts data of the transcription group were used for difference analysis. *p* < 0.05 and | log2fc | > 0.5 were set to screen the genes upregulated in CRC. The “ggplot2” package was used to create heat and volcano maps. Pan-cancer expression levels of SLC35A2 were analyzed using the TIMER database. The expression of methylation site cg26807389 related to SLC35A2 was analyzed through the website (https://biit.cs.ut.ee/methsurv/).

### Least absolute shrinkage and selection operator (LASSO)

To refine the model, some coefficients can be compressed and others can be set to zero by using a penalty function. Subset contraction is retained and a biased estimation is provided for processing data with complex collinearity. As a result of the use of this method, we were able to identify genes that are significantly associated with CRC diagnosis.

### Expression and prognosis analysis of gene SLC35A2 among different clinical groups

Based on UCSC database, SLC35A2 expression in colorectal cancer tissues and its correlation with the clinical characteristics of colorectal cancer patients were analyzed. By using the rank sum test, we investigated SLC35A2 expression gene in CRC and normal tissues, as well as the variation in that expression with sex, age, pathological stage, T, N and M stage. Using the log-rank test, a survival curve was drawn for patients with CRC to assess SLC35A2’s impact on the prognosis. The prognostic independent factors affecting the overall survival rate of CRC patients were evaluated using univariate and multivariate COX regression analyses. Based on Protein Atlas database (https://www.proteinatlas.org/), the immunohistochemical expression of SLC35A2 in normal colon tissue and colorectal cancer tissue was analyzed.

### Immuno-infiltration analysis

The degree of immune infiltration in samples from patients with CRC was determined by using the CIBERSORT method. Rank sum tests were used to investigate differences in immune cell infiltration between groups, and the Spearman method was used to look at the association with immune cells and the expression SLC35A2.

### GO and KEGG analysis

GO is an online database created by the Gene Ontology Consortium. The purpose of this database is to build a database that defines and describes the function of genes and proteins and applies it to all kinds of species. There are three categories: BP, CC and MF. KEGG is a database that analyzes gene functions systematically and connects genomic and functional information, including metabolic pathway, hierarchical classification, gene, and genome databases. Enriched pathways were obtained by comparing the studied genes with the pathway gene set in the database.

### Construct nomogram

To enhance the evaluation of patients’ prognosis, the clinical data and SLC35A2 gene expression were combined. A nomogram was designed to predict patient mortality at 1, 3 and 5 years.

### Clinical tissue samples from patients

Patients receiving routine surgical treatment at Fuyang Hospital affiliated with Anhui Medical University between 2021 and 2022 were given colorectal cancer tissues and matched para-cancerous tissues. All cases were confirmed by pathology and matched with normal tissues, with a total of 60 cases. We have 37 cases of colon cancer and 23 cases of rectal cancer. Neither radiotherapy nor chemotherapy were administered to these patients before surgery. After the operation, the tissue was fixed in 10% neutral formalin and prepared for immunohistochemical staining. The research was carried out in accordance with the Helsinki Declaration. All the cases were approved by Fuyang Hospital affiliated with Anhui Medical University’s Academic Committee. All study participants received written informed consent.

### Immunohistochemical analysis

The experiment was carried out by immunohistochemical manual kit (Maixin Biotechnologies, Fuzhou, China). The tissue slices of 2.5 μm thick were prepared by the traditional method of dehydration, transparency, and paraffin embedding. The slices were dried in the oven at 60°C for 2 hours. These were placed in dimethyl benzene for chip dewaxing for 3 × 5 min, and hydrating for 3 × 5 min with alcohol concentration gradient from high to low. It was slowly rinsed with PBS, repaired with a sodium citrate solution at high pressure for 5 min, and then cooled automatically at 25°C. PBS buffer was used for 3 × 3 min, endogenous peroxidase was dripped into tissues for incubation for 10 min, the PBS buffer was used for 3 × 3 min, and SLC35A2 (13657-1-AP, Proteintech Group Inc., IL, USA) was diluted to 1:100. After the addition of the first antibody in the form of drops, the slices were incubated for 4 hours at room temperature and then washed for 3 × 3 minutes in PBS solution. After incubating at room temperature for 20 min, the reaction enhancing solution was rinsed with buffer solution for 3 × 3 min, the secondary antibody IgG solution was slowly added as drops, then incubated for 2 h at room temperature, and then rinsed with buffer solution for 3 × 3 min. The prepared DAB chromogenic solution was added, the color development was stopped after 4 min, and washed slowly with running water. Hematoxylin solution was used to re-dye the tissue sections for 6 minutes, after which they were subjected to anti-blue treatment in tap water for 15 min, dehydrated with a gradient of alcohol concentration from low to high, then rendered transparent in dimethylbenzene solution; a microscope was used to observe the expression of SLC35A2 on the sealed film after it was sealed with neutral resin. It was interpreted by two pathologists based on the staining results. Scoring of staining positive percentage was: <5% = 0, 5–25% = 1, 26–50% = 2, and > 50% = 3. Dyeing intensity scores ranged from 0 (no signal colour) to 2 (pale yellow and deep brown). The final immunohistochemical score was calculated by multiplying the scores of “staining positive percentage” and “dyeing intensity”: 0–1 = negative, 2–3 = weakly positive, 4–5 = moderate positive, 5–6 = strong positive [23]. Negative and poor positive results were regarded as low, while medium and high positive results were regarded as high levels of expression [24].

### Cell culture

NCM460 cells were purchased from Wuhan Procell Life Technology Co. Ltd. (Wuhan, China), the American Tissue Culture Collection Company (VA, USA) provided the HCT116 and LOVO cells. NCM460 cells were cultured in a high sugar medium of DMEM containing 10% fetal bovine serum and 5% double antibody, while LOVO and HCT116 cells were cultured in 1640 medium containing 10% fetal bovine serum and 5% double antibody, the cells placed in an incubator at 37°C and 5% CO2.

### Western blotting

Intestinal epithelial cells (NCM460) and CRC cells (HCT116 and LOVO) were collected, and total proteins were extracted using RIPA lysate then separated using 6% SDS polyacrylamide gel electrophoresis. The primary antibodies SLC35A2 (dilution ratio 1:1000) and GAPDH (dilution ratio 1: 1000) were added, incubated overnight in a refrigerator at 4°C, and the corresponding HRP labeled secondary antibody (dilution ratio 1:10000) was diluted with TBST and incubated at room temperature for 2 h. Finally, a gel imager (Bio-Rad, CA, USA) was used for the exposure and recording.

### qRT-PCR

In NCM460 and CRC cells, total RNA was extracted using TRIzon reagent (Ambion, TX, USA) according to the manufacturer’s instructions. A reverse transcription kit (Takara, Otsu, Japan) was used for the synthesis of complementary DNA (cDNA). The cDNA was amplified using 2 × SYBR Green PCR Master Mix (Life Technologies, Xiamen, China). An internal control was conducted using GAPDH. Relative quantification was performed using the −2ΔΔCt method. IBM SPSS Statistics (version 20.0) was used to measure the relative expression of the two groups, and to assess the difference in the relative expression between the two groups. The specific primer sequences used in this experiment were as follows: SLC35A2: Forward: 5′-TCGCAGTGCCCTCTCTCAT-3′; Reverse: 5′-CAGTCCCCCTCCCTTGTGT-3′. GAPDH: Forward: 5′-TCAAGAAGGTGGTGAAGCAGG-3′; Reverse: 5′-TCAAAGGTGGAGGAGTGGGT-3′.

### Statistical analysis

The Deseq2 coating was applied to analyse the difference between CRC and normal tissues. Univariate and LASSO were used to select for genes related to prognosis. The effect of SLC35A2 on prognosis in CRC patients was investigated by univariate and multivariate Cox analysis. The Kaplan-Meier method was used for survival analysis. An analysis of SLC35A2 expression in CRC and other clinical groups was conducted using a rank sum test. The Spearman method was applied to analyze the relationship of immunity cells with SLC35A2. The correlation of SLC35A2 expression with clinicopathologic parameters of CRC was analyzed by the chi-square method.

The qRT-PCR and western blotting data were statistically analyzed using SPSS 22.0 software, and the quantitative results are expressed as the average standard deviation $(\bar{x}\pm \text{S}).$ An independent sample *t*-test was used to compare the two groups. Comparing quantitative numerical values between groups was carried out using a one-way ANOVA. *p* < 0.05 was defined as the difference with statistical significance.

### Data availability statement

The original contributions presented in this study are included in the article/Supplementary Materials, and further inquiries can be directed to the corresponding author.

## RESULTS

### Differential analysis of SLC family gene expression in CRC

**Figure 1:**
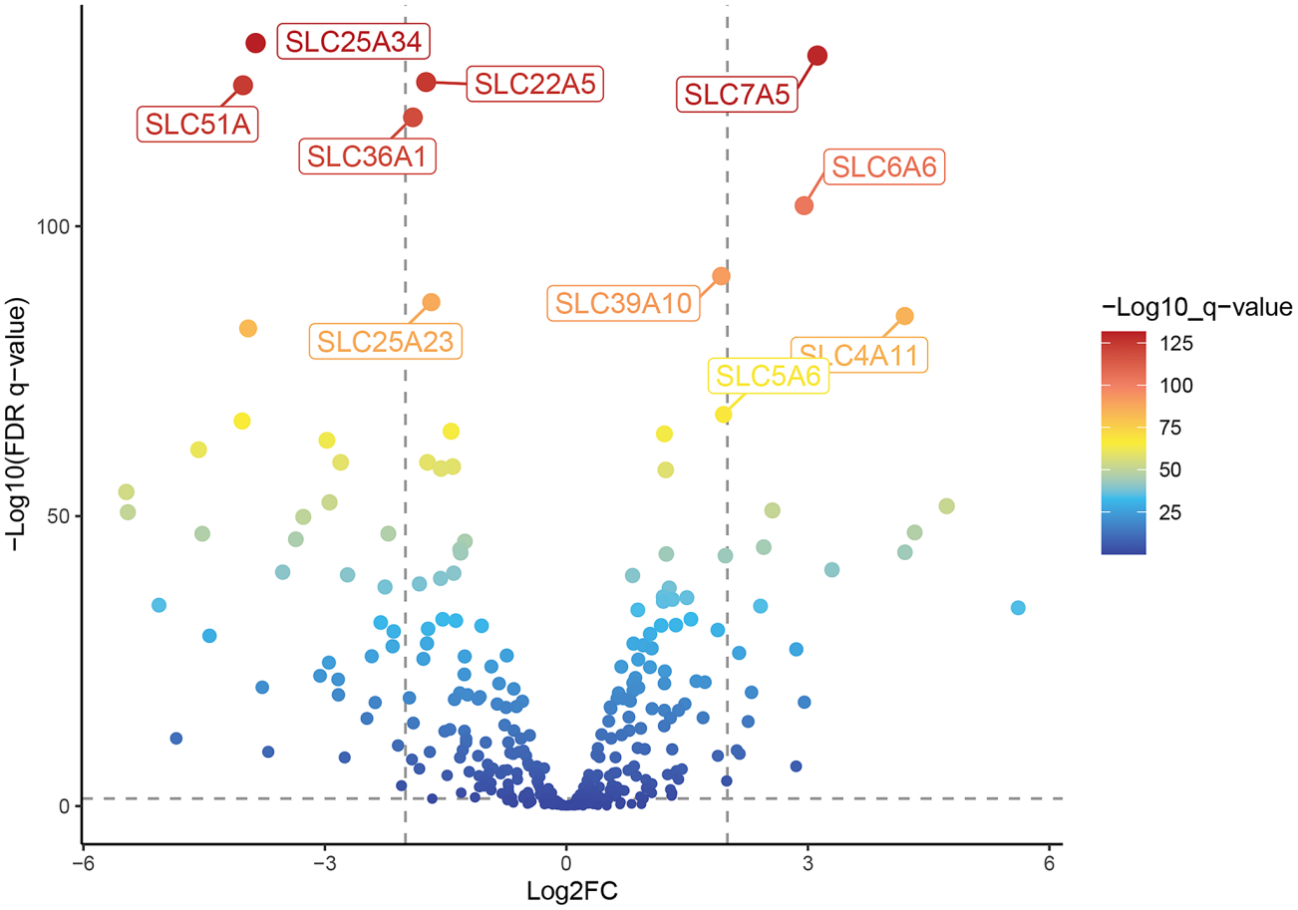
**Difference analysis of the human solute carrier (SLC) gene family between tumor tissue and adjacent normal tissue in patients with colorectal cancer.** The volcano map of 224 differentially expressed SLC family genes in colorectal cancer compared with normal tissues shows the top 5 up-regulated and down-regulated genes respectively.

Because data on the correlation between SLC family genes and CRC are limited, we first obtained the CRC transcriptome data HTSeq-Counts from the UCSC database, and then used the “Deseq2” package to analyze the differences between tumor tissues and adjacent normal tissues of CRC patients; we set *p* < 0.05, | log2fc | > 0.5, and obtained 107 upregulated genes and 117 downregulated genes. As shown in Supplementary Figure 1, there were 224 genes with different expression levels in the SLC family with respect to CRC. As shown in Figure 1, among the 224 genes with different expression levels, the top five genes with the most significant upregulation were SLC7A5, SLC6A6, SLC39A10, SLC4A11, and SLC5A6. The top five downregulated genes were SLC25A34, SLC22A5, SLC51A, SLC36A1, and SLC25A23.

### Relationship between the expression levels of SLC family members and CRC prognosis

**Figure 2:**
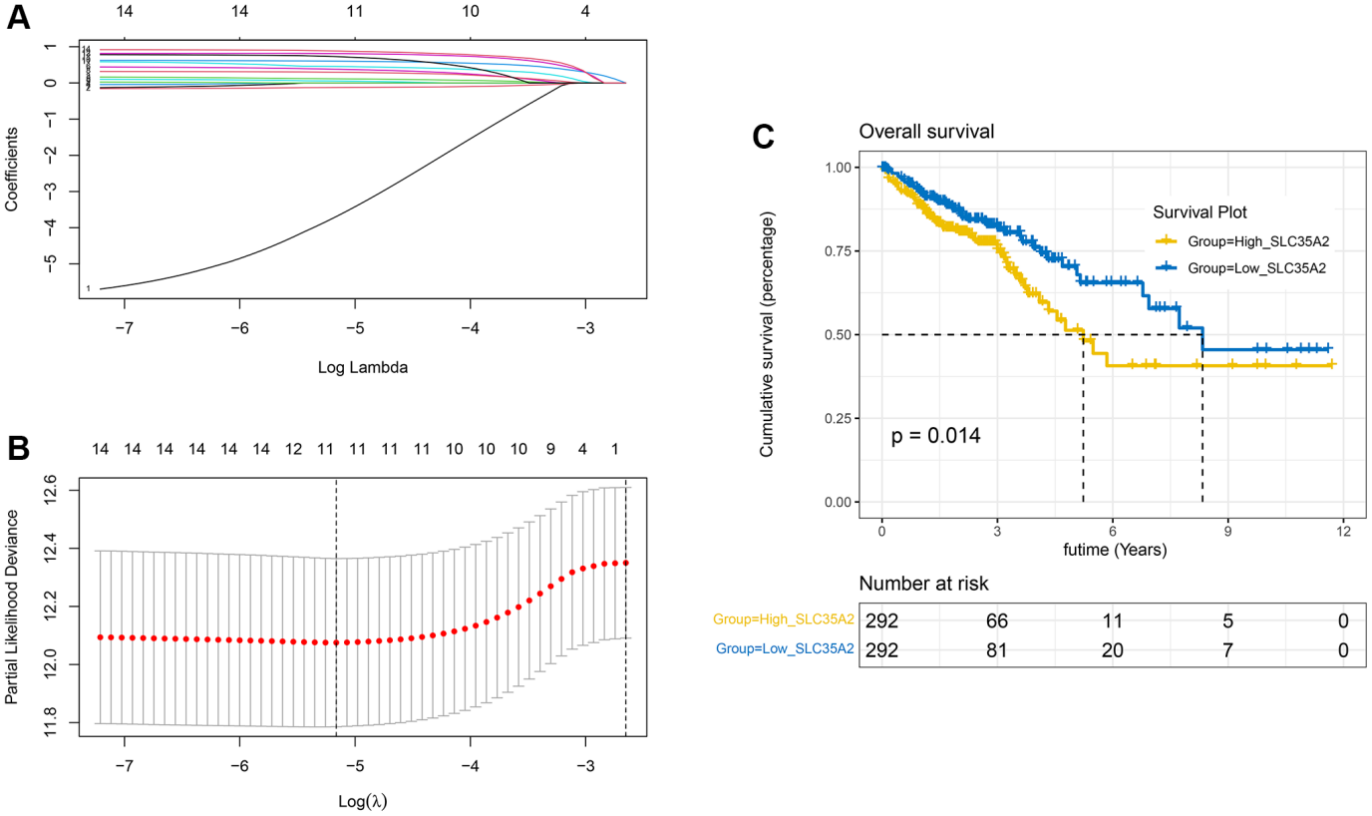
**The acquisition of the important gene SLC35A2 related to the prognosis.** (**A**, **B**) When the partial likelihood deviation reaches a minimum, the optimal λ is determined and the LASSO coefficients of the most useful prognostic genes are further generated. (**C**) Survival analysis of TCGA database cohort. The results indicated that high expression of SLC35A2 was related to poor prognosis in colorectal cancer (*p* = 0.014).

To find out more diagnostic markers and genes that affect the prognosis of colon cancer patients, the above-mentioned 107 upregulated genes were first analyzed by univariate Cox, with *p* < 0.05, and a total of 14 genes that influenced the prognosis of patients were obtained; LASSO analysis was performed. Setting the family parameter in glmnet function to “cox” and maxit to “1000”, it was found that when the best Lambda is 0.00572, there are 11 genes included (Figure 2A, 2B), which are SLC2A2, SLC12A2, SLC2A3, SLC12A9, SLC35A2, SLC4A2, SLC16A8, SLC6A1, SLC38A8, SLC24A2, and SLC30A3. Among these 11 genes, we found that only the upregulation of SLC35A2 had a substantial relationship with poor prognosis in CRC patients. The median expression of SLC35A2 was used to classify patients with TCGA CRC into high-expression and low-expression groups, and the difference in survival time was measured. The results indicated that the elevated level of SLC35A2 was associated with poor prognosis in CRC (*p* = 0.014) (Figure 2C), while other genes were not associated with prognosis and had no statistical significance (*p* > 0.05).

### The pan-cancer expression of SLC35A2

**Figure 3:**
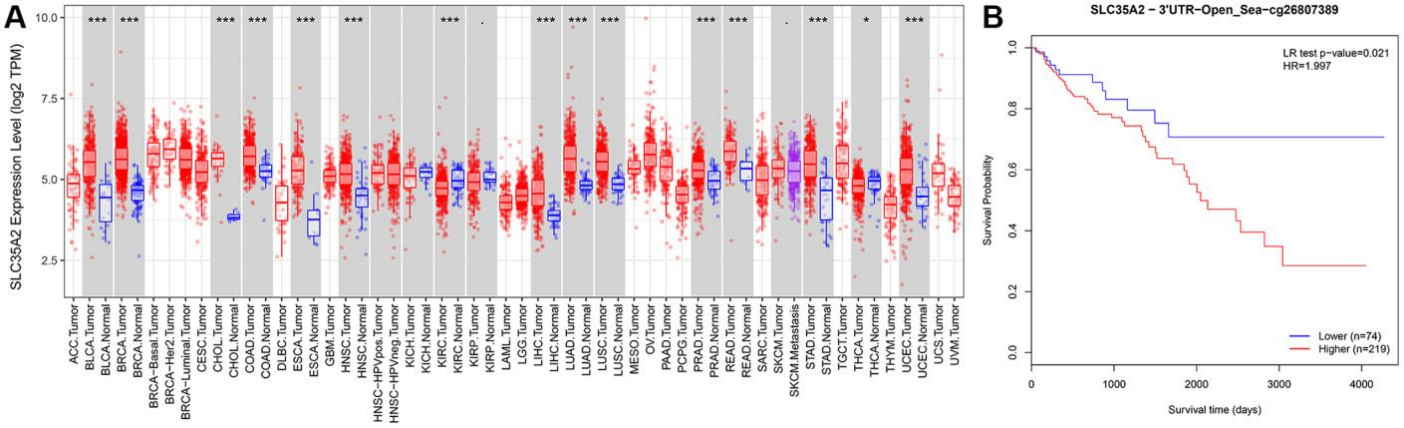
**Analysis of pan-cancer SLC35A2 expression and survival analysis of methylation site cg26807389 related to SLC35A2 with CRC patients.** (**A**) Differential expression of SLC35A2 was found in various tumor and normal tissues in TIMER database. Among them, the expression of SLC35A2 was elevated in colorectal adenocarcinoma (COAD) and rectal adenocarcinoma (READ). (^*^p < 0.05, ^**^p < 0.01, ^***^p < 0.001). (**B**) Survival analysis of high expression group of methylation site cg26807389. The results showed that CRC patients with high expression of methylation site cg26807389 had poor prognosis (*p* = 0.021).

We used the TIMER database to explore the expression of SLC35A2 in pan-cancer and entered the gene name SLC35A2 into the DiffExp module of this website to determine its expression in human tumors and adjacent normal tissues. As shown in Figure 3, SLC35A2 was differentially expressed in different types of cancers, including BLCA, BRCA, CHOL, COAD, ESCA, HNSC, KIRC, LIHC, LUAD, LUSC, PRAD, READ, STAD, UCEC (*p* < 0.001), and THCA (*p* < 0.05). The high expression of SLC35A2 in many tumors shows that SLC35A2 may be a tumor-related gene. It can be seen from the figure that SLC35A2 is upregulated in COAD and READ, but downregulated in KIRC and THCA.

Then we analyzed the expression of methylation site cg26807389 related to SLC35A2 through the website (https://biit.cs.ut.ee/methsurv/), and divided the CRC patients into high-expression and low-expression group. The results showed that the high expression of methylation site cg26807389 related to SLC35A2 was related to poor prognosis (*p* < 0.05).

### Correlation between SLC35A2 expression in CRC and clinicopathological features

**Figure 4:**
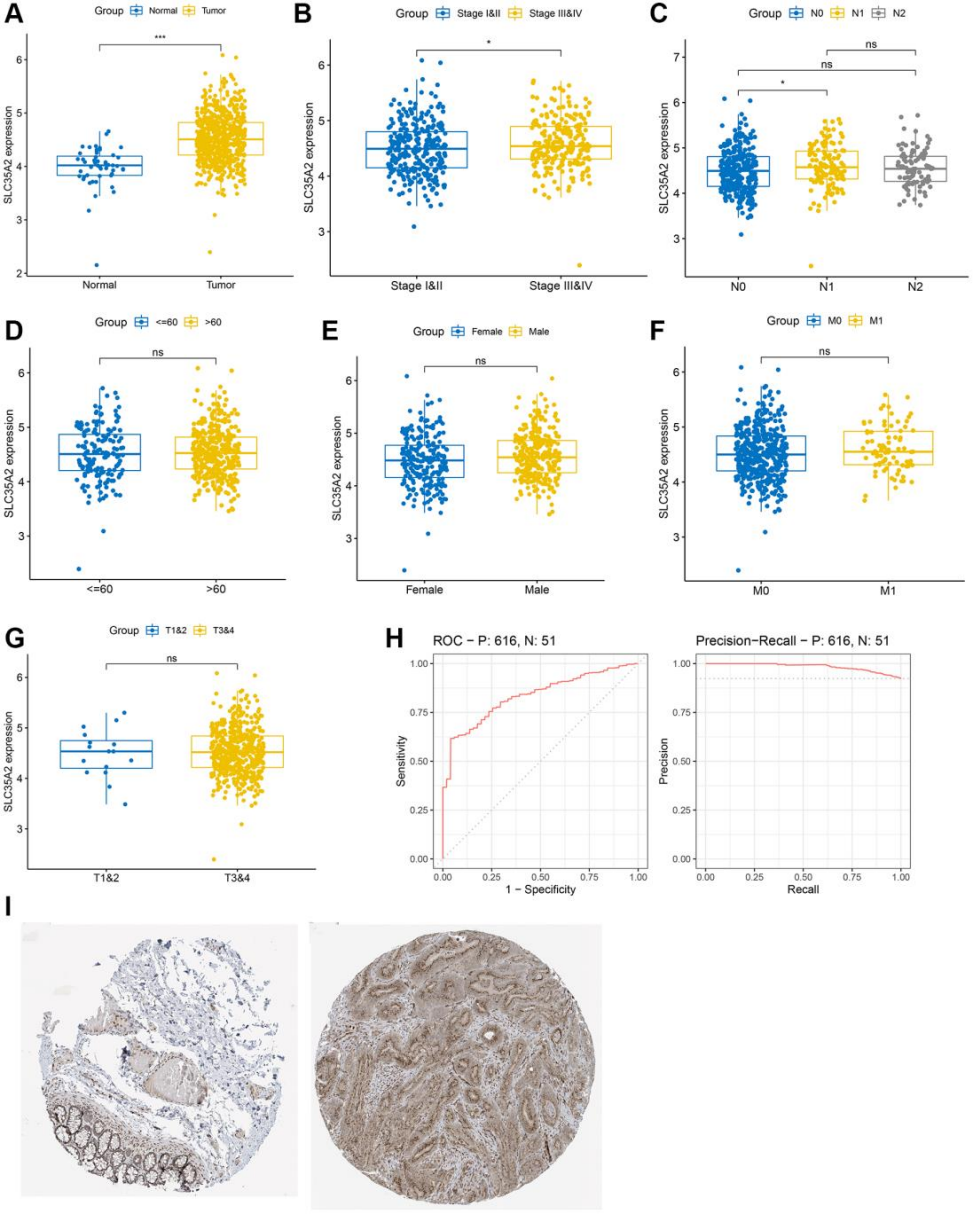
**The relationship of SLC35A2 expression and clinical features in patients with colorectal cancer.** (**A**) The expression of SLC35A2 in colorectal carcinoma was higher than that of normal tissues. (**B**) SLC35A2 expression was higher in Stages III and IV than in Stages I and II. (**C**) The difference of SLC35A2 expression between N0 and N1 was statistically significant (*p* < 0.05). (**D**–**G**) The expression level of SLC35A2 in different sexes, age, M and T stages was not statistically significant (*p* > 0.05). (**H**) The diagnostic value of SLC35A2 in CRC was assessed by drawing ROC and PRC curves. The AUC of the ROC curve is 0.834 and the PRC curve is 0.984 (^*^p < 0.05, ^**^p < 0.01, ^***^p < 0.001, ns means *p* > 0.05). (**I**) Representative images of IHC staining of SLC35A2 in normal colon tissue (left) and colorectal cancer tissue (right) was presented.

Based on the UCSC database, the relationship between SLC35A2 expression and the clinical features of CRC patients was analyzed. As illustrated in Figure 4A, SLC35A2 expression in CRC tissues was significantly higher than that in adjacent normal tissues (*p* < 0.001), and SLC35A2 was found to be higher in tumor pathological stages III and IV than in stage I and stage II (*p* < 0.05, Figure 4B). The expression of SLC35A2 was analyzed according to lymph node metastasis status, and it was found that there was a difference between N0 and N1 stages (*p* < 0.05, Figure 4C). In addition, we found that the expression of SLC35A2 was independent of age, and there was no significant difference between CRC patients aged > 60 years and CRC patients aged ≤ 60 years (Figure 4D). Similarly, we also found that there was no significant difference in the expression of this gene between male and female CRC patients in terms of gender (Figure 4E). In T and M staging, there was no significant difference in the expression level of SLC35A2 between tumor tissues and adjacent normal tissues (Figure 4F, 4G). In summary, our research showed that the expression of SLC35A2 is related to tumor stage and lymph node metastasis. In addition, we evaluated the diagnostic value of SLC35A2 in CRC by Receiver Operating Characteristic (ROC) and Precision Recall Curve (PRC) curves (Figure 4H). The results showed that the Area Under Curve (AUC) value of the ROC curve was 0.834 and that of the PRC curve was 0.984, these results suggest that SLC35A2 might potentially be a useful CRC diagnostic marker. In addition, we analyzed the immunohistochemical expression of SLC35A2 in normal colon tissue and colorectal cancer tissue in Protein Atlas database (https://www.proteinatlas.org/) (Figure 4I), and found that SLC35A2 protein was highly expressed in colorectal cancer tissue.

We also used univariate and multivariate Cox regression analyses to determine the prognostic value of SLC35A2 expression (Table 1). In univariate Cox regression analysis, the low survival rate was associated with elevated expression of SLC35A2 (HR = 1.426, 95% CI = 1.007–2.020; *p* = 0.046), which was significantly correlated with T4 stage (HR = 6.151, 95% CI = 1.458–25.953; *p* = 0.013), N1 stage (HR = 1.774, 95% CI = 1.131–2.781; *p* = 0.013), N2 stage (HR = 3.873, 95% CI = 2.588–5.796; *p* < 0.001), M stage (HR = 3.989, 95% CI = 2.684–5.929; *p* < 0.001), pathological stage III (HR = 3.263, 95% CI = 1.462–7.283; *p* = 0.004), pathological stage IV (HR = 8.065, 95% CI = 3.605–18.043; *p* < 0.001), age (HR = 1.939, 95% CI = 1.320–2.849; *p* < 0.001). In multivariate Cox regression analysis, there was a significant correlation between low survival and high level of SLC35A2 (HR = 1.548, 95% CI = 1.049–2.285; *p* = 0.028), N1 stage (HR = 0.272, 95% CI = 0.097–0.763; *p* = 0.013), M stage (HR = 22.626, 95% CI = 4.063–125.999; *p* < 0.001), pathological stage III (HR = 12.509, 95% CI = 2.118–73.860; *p* = 0.005), and age (HR = 3.041, 95% CI = 1.923–4.808; *p* < 0.001). Our results show that SLC35A2 overexpression, N1 stage, and pathological stage III are independent risk factors for poor prognosis of CRC patients.

**Table 1:** Univariate analysis and multivariate analysis on overall survival in colorectal cancer patients.

**Characteristics**		**Univariate Cox analysis**		**Multivariate Cox analysis**	
		**Hazard ratio (95% CI)**	***P* value**	**Hazard ratio (95% CI)**	***P* value**
SLC35A2 (High vs. Low)		1.426 (1.007–2.020)	0.046^*^	1.548 (1.049–2.285)	0.028^*^
T stage	T2 vs. T1	1.000 (0.216–4.639)	1.000	0.581 (0.062–5.470)	0.635
	T3 vs. T1	2.047 (0.504–8.317)	0.316	0.708 (0.057–8.771)	0.788
	T4 vs. T1	6.151 (1.458–25.953)	0.013^*^	1.750 (0.138–22.209)	0.666
N stage	N1 vs. N0	1.774 (1.131–2.781)	0.013^*^	0.272 (0.097–0.763)	0.013^*^
	N2 vs. N0	3.873 (2.588–5.796)	<0.001^***^	0.531 (0.195–1.445)	0.215
M stage (M1 vs. M0)		3.989 (2.684–5.929)	<0.001^***^	22.626 (4.063–125.999)	<0.001^***^
Pathologic stage	Stage II vs. Stage I	1.735 (0.765–3.933)	0.187	2.086 (0.390–11.164)	0.390
	Stage III vs. Stage I	3.263 (1.462–7.283)	0.004^**^	12.509 (2.118–73.860)	0.005^**^
	Stage IV vs. Stage I	8.065 (3.605–18.043)	<0.001^***^		
Gender (Male vs. Female)		0.949 (0.671–1.344)	0.769		
Age (>65 vs. ≤65)		1.939 (1.320–2.849)	<0.001^***^	3.041 (1.923–4.808)	<0.001^***^

(^*^p < 0.05, ^**^p < 0.01, ^***^p < 0.001).

### Immuno-infiltration analysis

**Figure 5:**
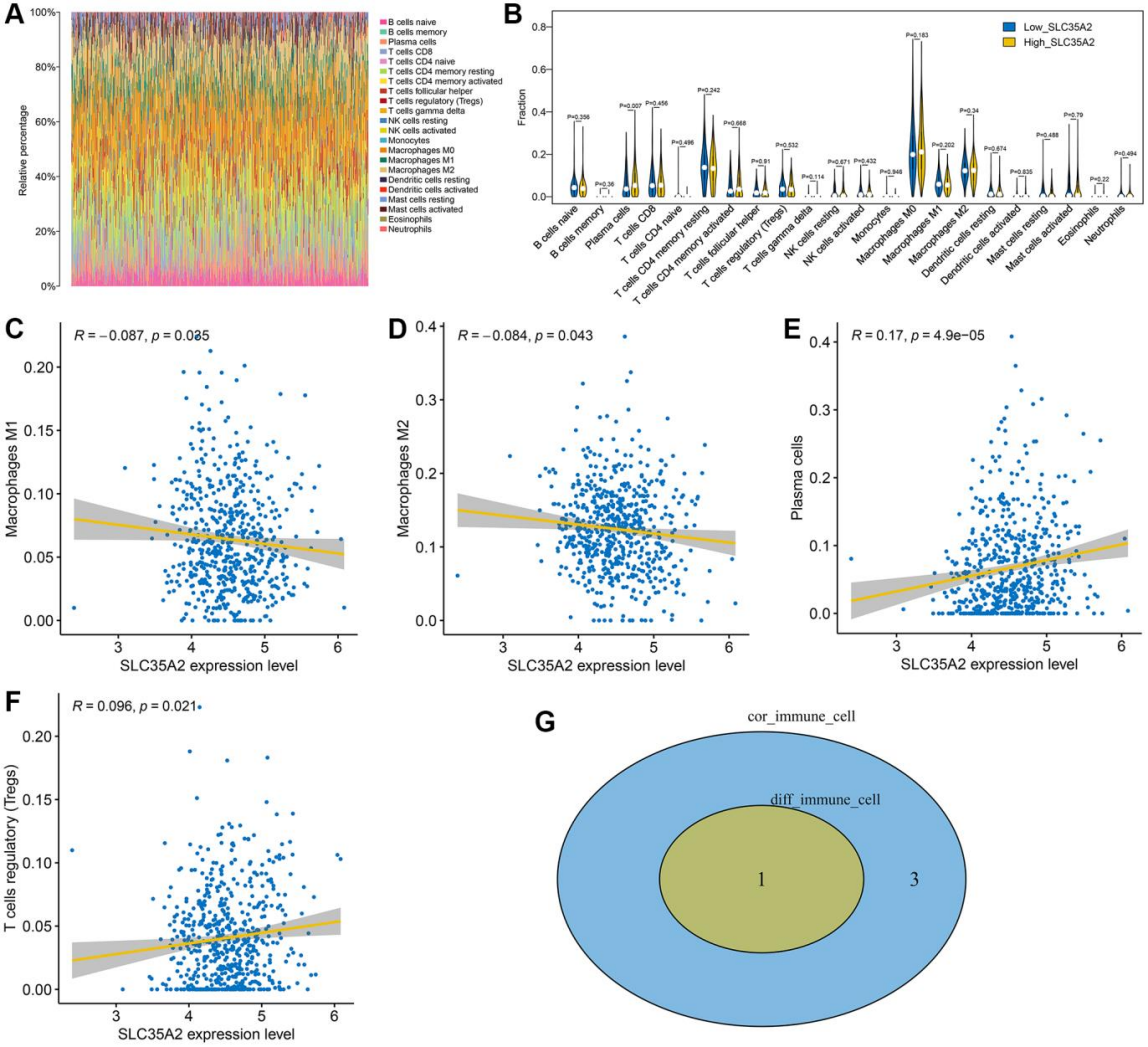
**Analysis of immune microenvironment of colorectal cancer.** (**A**) The overall immune cell infiltration of CRC patients. (**B**) The high-SLC35A2-expression and low-SLC35A2-expression groups differed in the level of immune cell infiltration, and only one distinct immune cell was obtained: plasma cells. (**C**–**F**) Correlation analysis indicated that there was significant correlation between 4 types of immunity and SLC35A2: macrophages M1 and M2, T cell regulatory (Tregs) and plasma cells. (**G**) One of the most significant immune cell types was obtained: plasma cells.

The relationship between SLC35A2 and immune cells was further investigated using CIBERSORT to evaluate the infiltration of immune cells in CRC. Figure 5A shows the overall immune cell infiltration of CRC patients; plasma cells and T cells were found to be major immune cells in CRC. CRC samples were divided into a SLC35A2 low-expression group and a SLC35A2 high-expression group. We studied changes in immune cell infiltration levels between the two groups. As shown in Figure 5B, plasma cell expression was upregulated in the SLC35A2 high-expression group (*p* < 0.05). We then analyzed the correlation between the expression of SLC35A2 gene and immune cells, and found that SLC35A2 gene was correlated with four kinds of immune cells (*p* < 0.05), among which the positive correlation immune cells were T cell regulatory (Tregs) and plasma cells (*p* < 0.05), while macrophages M1 and M2 (*p* < 0.05) were negatively correlated with the expression level of SLC35A2 (Figure 5C–5F). Finally, we crossed the immune cells obtained by the two methods and got the most significant immune cell: plasma cell (Figure 5G).

### GO/KEGG enrichment analysis

**Figure 6:**
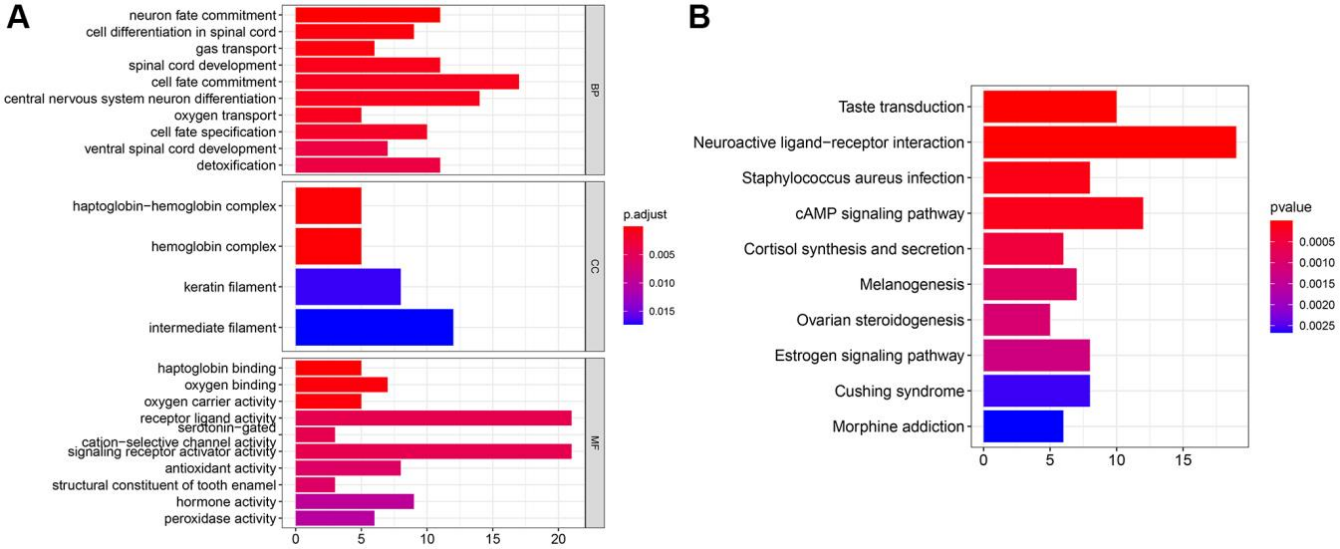
**GO/KEGG analysis of gene SLC35A2 in colorectal carcinoma.** (**A**) GO analysis of SLC35A2. The results were classified into Biological Process (BP), Cell Composition (CC) and Biological Function (MF). (**B**) KEGG analysis of SLC35A2 in colorectal tissues.

To further explore the role of SLC35A2, a GO/KEGG enrichment analysis was performed. According to the final results (Figure 6A), especially the analysis of CC, BP, and MF, SLC35A2 was related to cell fate commitment, central nervous system neuron differentiation, and neuronal fate commitment in BP. However, CC analysis also revealed that SLC35A2 is related to the haptoglobin-hemoglobin complex and hemoglobin complex, and MF analysis of GO showed that the main functions of SLC35A2 related genes were related to oxygen binding and haptoglobin binding. KEGG enrichment analysis (Figure 6B) showed that SLC35A2 is mainly involved in neuroactive ligand receptor interaction, cAMP signaling pathway, and taste transduction.

### A nomogram based on the SLC35A2 expression

**Figure 7:**
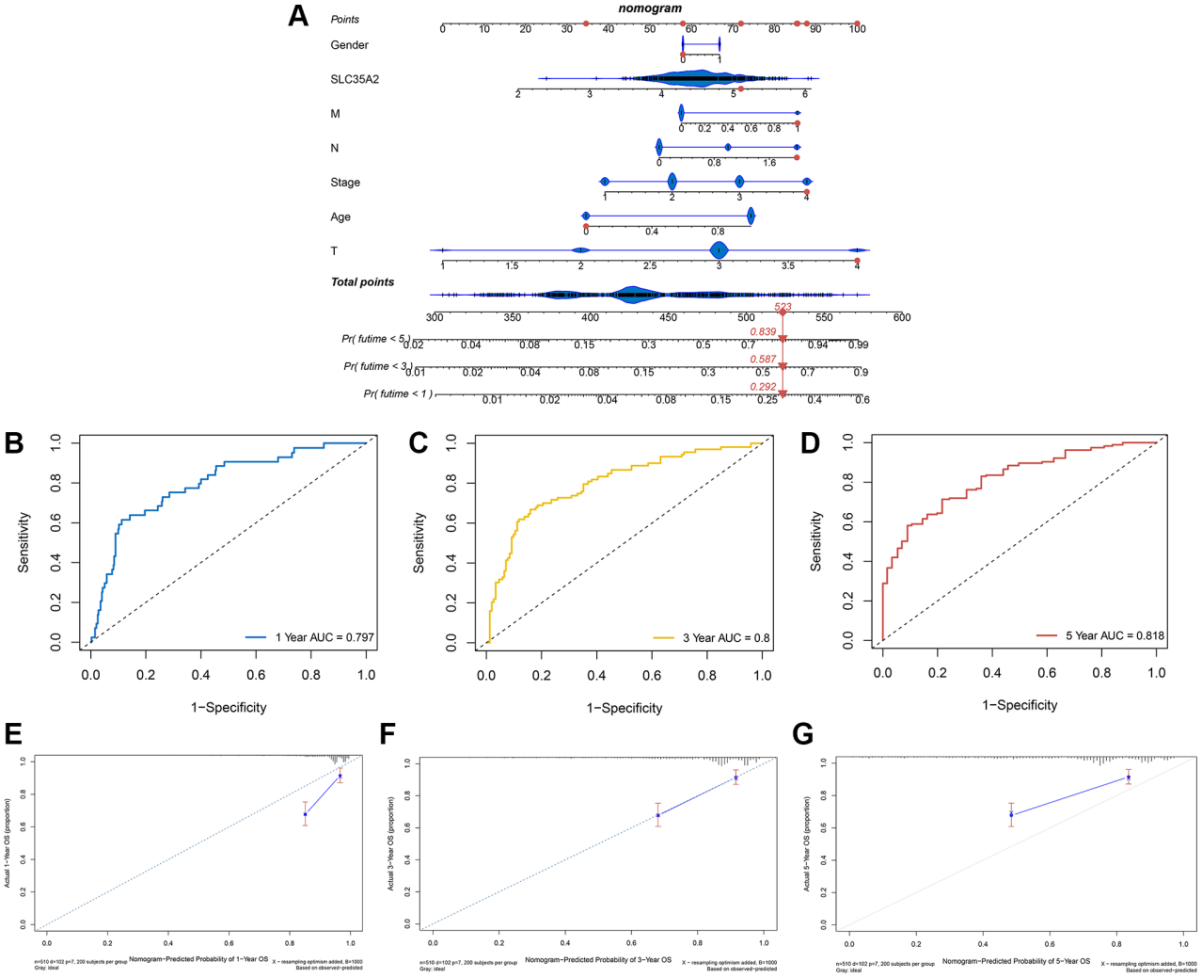
**Construction of a nomogram to predict prognosis of patients with colorectal cancer.** (**A**) Based on SLC35A2 expression and clinical characteristics of patients “TCGA-AA-A02K”, a nomogram was constructed. The 1, 3 and 5-years mortality rates of patient “TCGA-AA-A02K” were 0.292, 0.587 and 0.839, respectively. (**B**–**D**) The AUC of this nomogram with 1, 3 and 5-year ROC curves were 0.797, 0.8 and 0.818, respectively. (**E**–**G**) Calibration curves of 1, 3, and 5 years indicated that the nomogram was able to accurately predict colorectal cancer patients’ survival.

Based on SLC35A2 expression and clinical features of CRC patients, we constructed a nomogram to further evaluate the survival rate of CRC patients. As shown in Figure 7A, the 1-year, 3-year, and 5-year mortality rates of the patient “TCGA-AA-A02K” were 0.292, 0.587 and 0.839, respectively. The AUC values of the ROC curves at 1-year, 3-year and 5-year of this nomogram were 0.797, 0.8 and 0.818, respectively (Figure 7B–7D). According to the calibration curves of 1, 3 and 5 years (Figure 7E–7G), the nomogram can accurately predict the survival rate of patients with CRC.

### The expression of SLC35A2 in CRC tissues and cells

**Figure 8:**
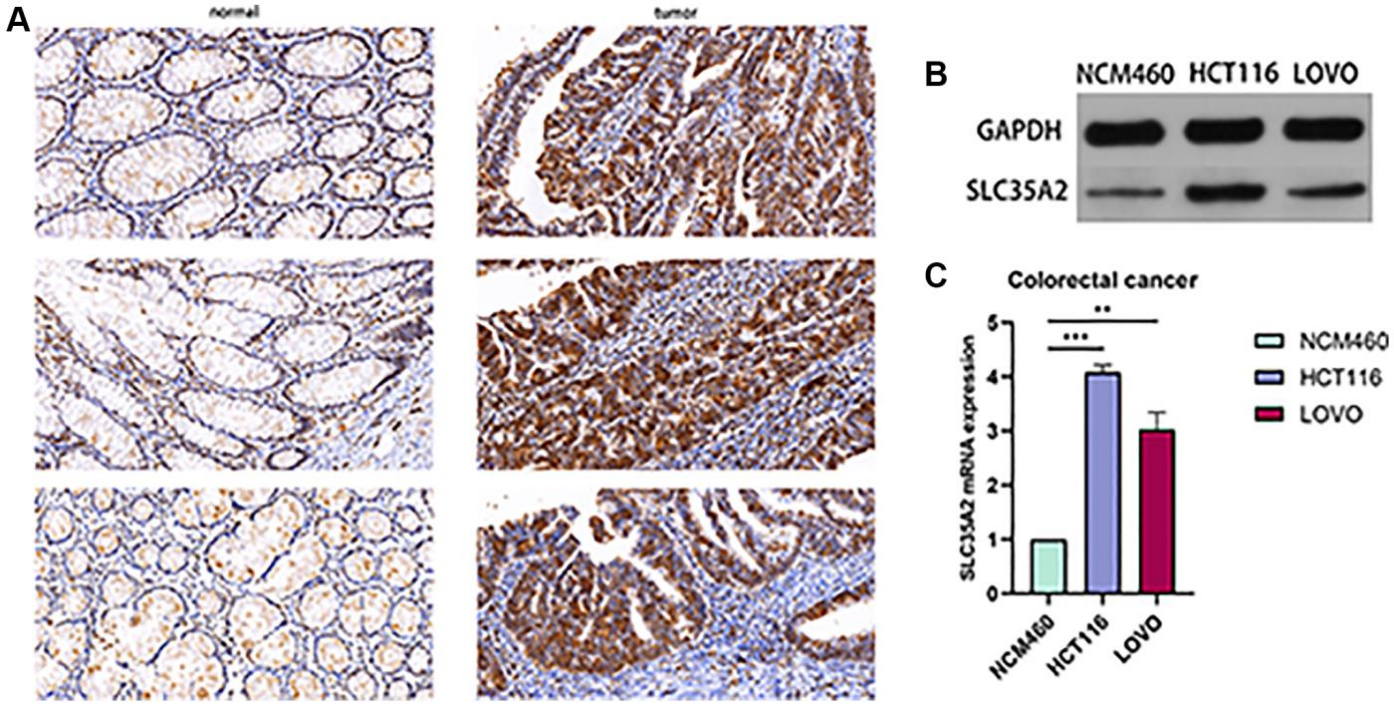
**Expression of SLC35A2 in colorectal cancer tissues and cells.** (**A**) Representative immunohistochemical staining of SLC35A2 in CRC tumor tissues and matched paracancerous tissues. Strong positive expression of SLC35A2 expression in CRC tissues, weak positive of SLC35A2 expression in matched paracancerous tissues. Scale bar = 50 μm. (**B**) Western blotting was used to detect the protein expression of SLC35A2 in intestinal epithelium (NCM460) and colorectal carcinoma (HCT116, LOVO). (**C**) qRT-PCR was used to validate SLC35A2 expression in colorectal carcinoma. (^**^p < 0.01, ^***^p < 0.001).

To further verify the SLC35A2 protein expression in CRC tissues, we stained paraffin sections of CRC tissues and adjacent tissues from 60 cases by immunohistochemistry (IHC). Similar to the analysis results in the protein database, the IHC image (Figure 8A) showed that the positive expression of SLC35A2 was mainly located in the nuclear membrane and cytoplasm, showing dark brown granules, highly positive expression was found in the CRC tissues, but was weakly expressed in adjacent normal tissues. Combined with the clinical and pathological features of CRC patients (Table 2), it was found that SLC35A2 was highly expressed in 78.33% (47/60) of cancer tissue samples, and low expression in 21.67% (13/60) of CRC tissue samples (*p* < 0.05). There was a significant correlation between the expression of SLC35A2 and pathological stage of the tumor (*p* < 0.05). Our western blotting experiments showed similar results. SLC35A2 protein expression in CRC (HCT116, LOVO) was significantly higher than that in the intestinal epithelial cell line NCM460 (Figure 8B). In addition, the relative expression level of SLC35A2 in CRC cell lines was quantitatively detected by qRT-PCR. The experimental results showed that SLC35A2 mRNA was overexpressed in HCT116 and LOVO compared with NCM460 (Figure 8C). Experimental results further confirmed that SLC35A2 was upregulated in CRC tissues and cell lines.

**Table 2:** Association between SLC35A2 expression and clinicopathological characteristics of colorectal cancer patients (*n* = 60).

**Features**	***n***	**SLC35A2 expression**		**χ2 value**	***P* value**
		**Low (*n* = 13)**	**High (*n* = 47)**		
Gender					
Male	32	5	27	1.475	0.225
Female	28	8	20		
Age (years)					
<60	37	7	30	0.111	0.739
≥60	23	6	17		
Size (cm)					
≤5.0	29	4	25	2.050	0.152
>5.0	31	9	22		
Pathologic stage					
I+II	15	7	8	4.472	0.034^*^
III+IV	45	6	39		
Differentiation					
High	8	1	7	0.654	0.721
Moderate	41	10	31		
Low	11	2	9		
Vascular invasion					
No	37	9	28	0.097	0.755
Yes	23	4	19		
Perineural invasion					
No	43	10	33	0.016	0.899
Yes	17	3	14		
T stage					
T1&2	28	7	21	0.344	0.558
T3&4	32	6	26		
N stage					
N0	32	9	23	2.124	0.346
N1	16	3	13		
N2	12	1	11
M stage					
M0	37	11	26	2.562	0.109
M1	23	2	21		

(^*^p < 0.05).

## DISCUSSION

Colorectal cancer is a global public health issue, and its incidence and mortality are increasing rapidly, causing a huge economic burden on patients and society [25]. Currently, surgical resection is the first choice for the treatment of CRC, supplemented with chemotherapy, radiotherapy, targeted therapy, and immunotherapy [26]. Unfortunately, in patients diagnosed with metastatic colorectal cancer, the postoperative survival rate of CRC is not ideal, and it is still one of the most difficult diseases to treat [23]. Therefore, if reliable prognostic biomarkers of CRC can be found, “high-risk” CRC patients can be identified early, which is of great significance for early diagnosis and improvement of prognosis.

Gene SLC35A2 is located on chromosome Xp11.23 [15], and encodes a multi-channel membrane protein for transports uridine diphosphate (UDP)-galactose, namely UGT [27]. UGT can be divided into two splicing forms, UGT1 and UGT2, because of the difference in eight amino acid residues at the C-terminal. UGT1 is only located in Golgi apparatus, while UGT2 is located in both endoplasmic reticulum and Golgi apparatus [28]. Previous studies have pointed out that SLC35A2 is the only known UDP-galactose transporter (UGT) in mammals [15], its mosaic mutation code is mainly located in UGT1 of Golgi apparatus, which can lead to some diseases related to congenital glycosylation disorder [29], such as congenital glycosylation disease (CDG) and isolated focal cortical dysplasia (IFCD) [30]. Most patients suffer from different degrees of nerve damage such as mental disability, deformity, and epileptic encephalopathy [28]. As an important member of NSTs family, SLC35A2 has specific UDP-galactose transporter activity and carbohydrate-proton co-transporter activity, which is mainly responsible for transporting UDP-galactose from the cytoplasm to Golgi vesicles, and glycosyltransferases play a role in Golgi vesicles to generate glycosyl [13]. NSTs is mainly involved in glucose metabolism and play a key role in the malignant biological behavior of cancer, which is conducive to the survival, proliferation, invasion, and metastasis of cancer cells [31]. Therefore, it has become key to understanding this membrane protein family in cancer metabolism research [14], which further indicates that SLC35A2 might play an important role in cancer development and cancer metabolism. In addition, SLC35A2 is strongly amplified after treatment with the DNA damage agent cisplatin, and plays a major role in the sensitivity of cisplatin in tumor therapy [32, 33]. To date, research on the biological function of SLC35A2 in tumors remains limited. This is, as far as we know, the first evidence that SLC35A2 is highly expressed in CRC. In this study, we used a number of bioinformatics databases to investigate the potential molecular mechanisms and their role in the prognosis of CRC.

In this research, we first obtained the CRC transcriptome data HTSeq-Counts from the UCSC database, compared the expression levels of SLC family genes in tumor tissues of patients with CRC and adjacent normal tissues, and analyzed the differences; we initially screened out 224 genes with different expression levels, of which 107 genes were upregulated. Then, 107 genes were analyzed by univariate analysis and LASSO, and 11 genes were further screened. Among them, only the increase in SLC35A2 expression was associated with poor prognosis in CRC (*p* = 0.014). The above results suggest that overexpression of SLC35A2 is might be an independent risk factor for poor prognosis in CRC patients. Subsequently, we analyzed SLC35A2 pan-cancer expression using the TIMER database and discovered that SLC35A2 was upregulated in COAD and READ. It is further proved that SLC35A2 is significantly overexpressed in CRC and is independently related to OS difference in CRC patients.

The purpose of this study was to investigate the clinical significance of SLC35A2 in CRC. In online database research, high SLC35A2 expression was observably associated with high tumor stage and lymph node metastasis. In addition, through analysis of the ROC curve, it was observed that SLC35A2 had good performance in distinguishing CRC tissue from non-CRC tissue (AUC = 0.834). Subsequent PRC curve analysis confirmed this conclusion (AUC = 0.984). Furthermore, univariate and multivariate Cox regression analysis showed that SLC35A2 was overexpressed (HR = 1.548, 95% CI = 1.049–2.285; *p* = 0.028), N1 (HR = 0.272, 95% CI = 0.097–0.763; *p* = 0.013), and pathological stage III (HR = 12.509, 95% CI = 2.118–73.860; *p* = 0.005) was independently correlated with OS in CRC patients. In this research, we also discovered that the SLC35A2 expression in CRC was higher than that of adjacent normal tissues. Then, clinical parameters of CRC patients were analyzed, and it was indicated that there was a significant correlation between SLC35A2 expression and pathologic stage (*p* < 0.05). The expression of SLC35A2 had no significant correlation with age, sex, differentiation degree, vascular nerve invasion, or T, N, and M stages (*p* > 0.05). In summary, the evidence suggests high expression of SLC35A2 in CRC tissues. Overexpression of SLC35A2 is an independent prognostic factor in postoperative patients with CRC, and may play a role in carcinogenesis and malignant progression. Its upregulation is related to the pathological stages of CRC, and it may serve as a potential diagnostic biomarker and predictor of poor prognosis.

Tumor cells inhibit the immune checkpoint pathway mediated by immune cells, which results in immune escape [34]. The discovery of tumor immune checkpoints has provided a new approach for tumor immunotherapy. Immune checkpoint blocking therapy is based on programmed death receptors and their ligands to enhance the host immune system’s ability to attack tumor cells by inhibiting tumor cell binding [35]. Therefore, the study of the CRC immune microenvironment is of great importance. Our research provides an immune landscape for CRC that can directly determine the abundance of each immune cell. Moreover, we compared the difference in the infiltration level of immune cells between the two groups of SLC35A2, identified the 4 types of immune cells that were most closely related to SLC35A2, and obtained the immune cells that were significantly related to SLC35A2: plasma cell factor. These results indicate that SLC35A2 can influence the immunological microenvironment of CRC by up-regulating the expression of plasma cells. It is worth further research. In addition, through GO/KEGG enrichment analysis, it was found that SLC35A2 has been found to participate in the interaction of neuroactive ligand-receptor and cAMP signaling pathway. However, the specific regulatory mechanism of SLC35A2 in CRC remains unclear. The cAMP pathway mediates the intracellular response to various hormones and neurotransmitters and is an important signaling pathway in tumor occurrence and development [36]. This may be the mechanism by which SLC35A2 performs many key biological functions in CRC.

In conclusion, the present study suggests that SLC35A2 is up-regulated in CRC tissues and cells, and the level of SLC35A2 is significantly related to the pathological stages of CRC patients, which may serve as prognostic markers in CRC and provide a forward-looking view for CRC diagnosis and therapy. Although we have analyzed and integrated some information from multiple databases and verified the high levels of SLC35A2 protein and mRNA in CRC through IHC, WB, and PCR experiments, there are still limitations in this study. Firstly, the SLC35A2 expression profile data was collected from many databases, and the absence of uniform standardization might result in inaccurate results. Second, we did not have enough animals to verify the results. In the future, more *in vivo* and *in vitro* studies will be required to validate our conclusions. The specific molecular mechanism of SLC35A2 promoting the occurrence and development of CRC still needs more follow-up experiments.

## CONCLUSION

In summary, as an oncogene, SLC35A2 is highly expressed in colorectal cancer tissues and cells. It can be used as a biomarker for the prognosis of colorectal cancer and may influence the prognosis of patients by influencing the tumor immune microenvironment.

## Supplementary Materials

Supplementary Figure 1
