# Supplementary material for: Comprehensive analysis of transcriptome data and experimental identification show that solute carrier 35 member A2 (SLC35A2) is a prognostic marker of colorectal cancer
Source: Aging (Albany NY). 2023 Oct 26;15(20):11554–70. doi: 10.18632/aging.205145 (PMC10637800; doi:10.18632/aging.205145)
Supplement: Supplementary Figure 1 [file aging-15-205145-s001.pdf]

SUPPLEMENTARY FIGURE

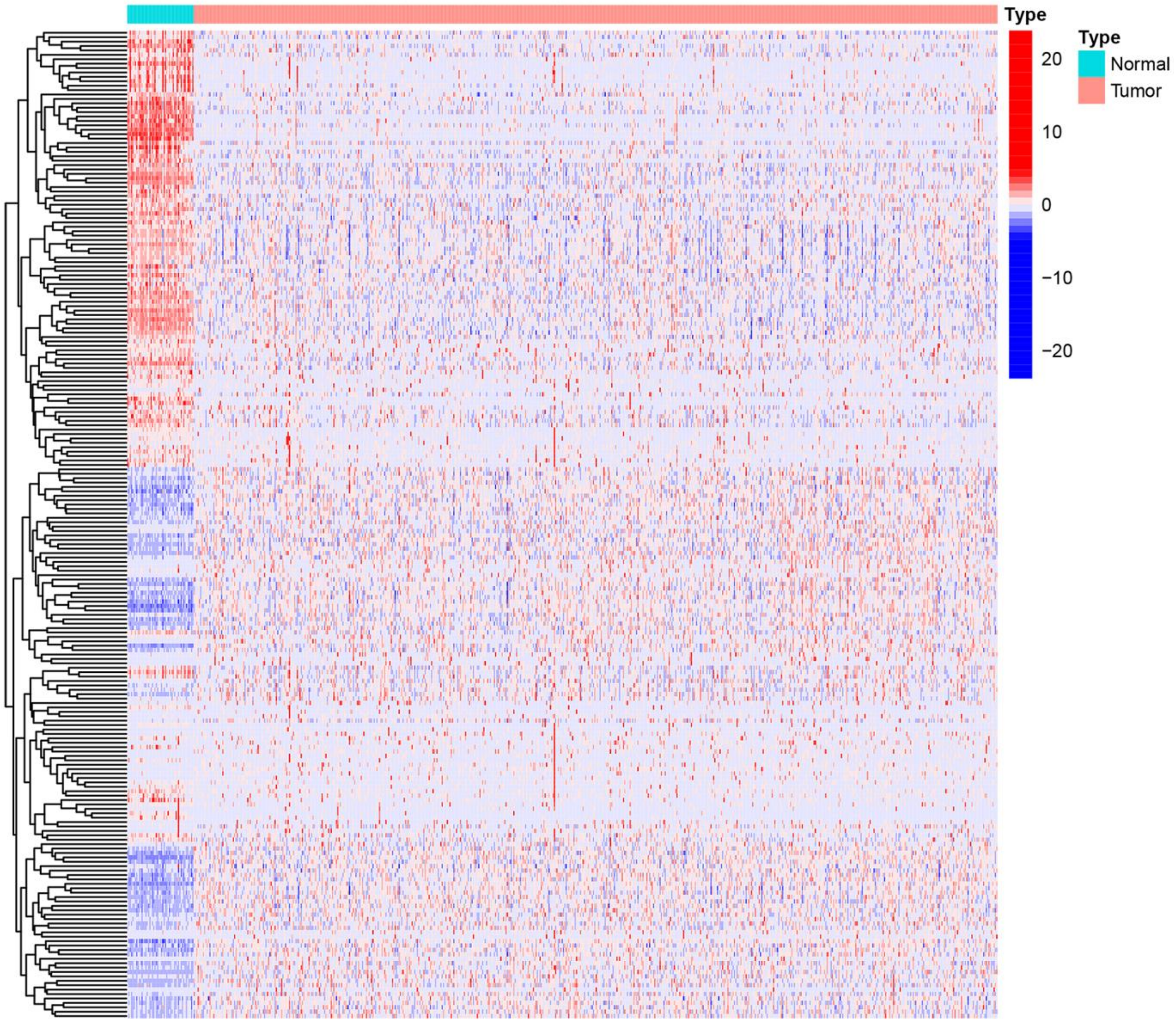

Supplementary Figure 1. The heat map of the expression of the 224 differentially expressed SLC family genes in colorectal cancer compared to normal tissue: the red represents the expression of upregulated genes, the blue represents the expression of downregulated genes. The darker the red color, the higher the expression level; the darker the blue color, the lower the expression level. See Annex 1 for specific gene information.
